# *Cblb*-deficient T cells are less susceptible to PD-L1-mediated inhibition

**DOI:** 10.18632/oncotarget.18360

**Published:** 2017-06-03

**Authors:** Sebastian Peer, Gottfried Baier, Thomas Gruber

**Affiliations:** ^1^ Department for Medical Genetics, Molecular and Clinical Pharmacology, Division of Translational Cell Genetics, Medical University of Innsbruck, Innsbruck, Austria

**Keywords:** immune checkpoints, cancer immunotherapy, T cells, Cbl-b

## Abstract

Modulation of the immune system for the treatment of primary and metastatic tumors has been a goal of cancer research for many years. The E3 ubiquitin ligase Cbl-b has been established as an intracellular checkpoint that limits T cell activation, critically contributing to the maintenance of self-tolerance. Furthermore, it has been shown that *Cblb* deficiency enhances T cell effector functions towards tumors. Blockade of the immune checkpoints CTLA-4 and PD-1/PD-L1 has recently emerged as a promising strategy in the development of effective cancer immune therapies. Therefore, we explored the concept of targeting different checkpoints concomitantly. Interestingly, we observed that CTLA-4 but not PD-L1 based immunotherapy selectively enhanced the anti-tumor phenotype of *Cblb*-deficient mice. In agreement with the *in vivo* results, *in vitro* experiments showed that *Cblb*^−/−^ T cells were less susceptible to PD-L1-mediated suppression of T cell proliferation and IFNγ secretion. Taken together, our findings reveal a so far unappreciated function of Cbl-b in the regulation of PD-1 signaling in murine T cells.

## INTRODUCTION

More than a century ago, Paul Ehrlich suggested that the immune system has a fundamental role in the rejection of cancer cells. He hypothesized that clinically detectable tumors would arise much more frequently if the immune system was not permanently eliminating transformed cells [1]. This concept generated vigorous debate, but decades of intense research confirmed that the immune compartment has indeed the potential to hold tumor growth in check. Accordingly, Paul Ehrlich's early suggestion was later refined to give rise to the immunosurveillance hypothesis of Burnet and Thomas [2], and more recently extended to the immunoediting concept by Schreiber and colleagues [3–5].

It is now evident that an effective immune response against tumors requires the activation of T lymphocytes, especially the cytotoxic CD8^+^ compartment. A number of tumor neoantigens have been described so far that are capable of eliciting an anti-tumor T cell response [6, 7]. Together with costimulatory signals, the recognition of such tumor antigens by the T cell receptor can lead to a profound activation of T cell effector functions that eventually are able to eradicate the tumor cells expressing this particular antigen [8].

On the other hand, it also became clear that inhibitory mechanisms counteract anti-tumor immunity. The so-called “immune checkpoints” help prevent autoimmunity, but also interfere with the attack of T cells against their tumor cell targets. Over the years, more and more regulatory pathways have been discovered that prevent successful anti-tumor T cell responses.

Two prominent members of the increasing group of immune checkpoint proteins are the T cell surface receptors CTLA-4 and PD-1.

Cytotoxic T-lymphocyte-associated protein-4 (CTLA-4) binds with high avidity to immunostimulatory CD80 and CD86 proteins on antigen-presenting cells and is thought to attenuate early T cell activation by sequestering its ligands away from the costimulatory receptor CD28 [9, 10]. CD80 and CD86 can even be removed from the surface of antigen- presenting cells by trans-endocytosis [11]. Furthermore, some reports suggest a T cell- intrinsic function of CTLA-4 by recruiting the protein phosphatases SHP2 and PP2A [12].

One study reported that CTLA-4 increased T cell motility, thereby preventing the formation of stable conjugates between T cells and antigen-presenting cells. This ultimately leads to decreased production of cytokines and T cell proliferation [13].

CTLA-4 also has a pivotal role in the activity of regulatory T cells: CTLA-4 triggering on T_regs_ enhances their immunosuppressive capacity [14, 15]. Recently it was shown that mature dendritic cells (DCs) secrete CTLA-4-containing microvesicles which can downregulate CD80/86 on bystander DCs, thereby negatively regulating T cell activation [16]. The fundamental role of CTLA-4 in maintaining immune homeostasis is demonstrated by the dramatic phenotype of *Ctla4* knockout mice. These mice exhibit lethal lymphoproliferative disease and multiorgan tissue destruction [17, 18].

Programmed cell death protein 1 (PD-1) primarily limits the activity of T cells in peripheral tissues and plays an important inhibitory role in the tumor microenvironment as tumor cells often express high levels of its ligands, PD-L1 and PD-L2 [19–22]. PD-1 mainly exerts its negative regulatory effect by recruiting SHP2 to its cytoplasmic tail [23]. As with CTLA-4, PD-1 engagement can enhance T cell motility by blocking the T cell receptor-mediated stop signal [24]. PD-1 is also expressed on regulatory T cells and can promote their induction and maintenance [25]. In comparison to *Ctla4* deficiency, the phenotype of *Pdcd1* knockout mice is relatively moderate, which might have important implications in the clinical application of the respective checkpoint inhibitors [26].

During the last two decades, the E3 ubiquitin ligase Cbl-b has emerged as an intracellular immune checkpoint. Cbl-b regulates T cell activation thresholds by mediating the requirement for CD28 costimulation, and loss of *Cblb* leads to anergy resistance and susceptibility to autoimmunity [27, 28]. Additionally, Cbl-b contributes to the maintenance of self-tolerance by mediating the immunosuppressive effects of TGFβ, and *Cblb*-deficient T cells are less sensitive to TGFβ and to inhibition by T_regs_ [29–32]. TGFβ secreted by tumor cells or tumor-infiltrating T_regs_ generates an immunosuppressive milieu that contributes to immune escape of tumor cells [33]. In this context, *Cblb* knockout mice display enhanced responses to a TGFβ-secreting tumor compared to wild-type mice [32]. In a number of studies it was demonstrated that *Cblb*^−/−^ mice can reject tumors in a T cell-dependent fashion, and the adoptive transfer of *Cblb*-deficient or *Cblb*-silenced CD8^+^ T cells together with a dendritic cell vaccine can significantly delay tumor growth and enhance survival rates [34–38]. Moreover, loss of *Cblb* or inactivation of its E3 ligase activity leads to rejection of metastatic tumors by natural killer cells [39].

The concept of using antagonists of inhibitory signals to enhance anti-tumor immune responses has found its way to the clinic with already promising results. Anti-CTLA-4 ipilimumab was the first “immune checkpoint” inhibitor that led to tumor regression and a survival benefit for patients with advanced melanoma and was therefore approved by the FDA in 2011 [40, 41].

Anti-PD-1 nivolumab was later also approved for the treatment of metastatic melanoma and a number of other cancer types. The combination of ipilimumab and nivolumab led to an improved survival benefit in metastatic melanoma patients in comparison to ipilimumab alone and was approved by the FDA in 2015 [42].

Targeting PD-1 signaling by blocking the PD-1 ligand PD-L1 is also a reasonable approach. For example, an anti-PD-L1 monoclonal antibody led to objective response rates of 6 - 17 % in melanoma, non-small-cell lung carcinoma, renal cell carcinoma, and ovarian cancer [43]. Anti-PD-L1 atezolizumab was approved by the FDA for the treatment of bladder cancer and non-small-cell lung cancer in 2016.

Nevertheless, the potency of these established checkpoint inhibitors is limited. For example, the efficacy of anti-CTLA-4 treatment depends on the immunogenicity of the tumor and can be dramatically enhanced by co-administration of a GM-CSF vaccine [44, 45]. Similarly, it has been suggested that the therapeutic benefit of PD-1 pathway blockade can be improved by combination with other approaches that induce antitumor responses [46].

Based on these data, we wanted to evaluate the efficacy of blocking PD-L1 or CTLA-4 in combination with loss of the intracellular checkpoint Cbl-b in a murine tumor model. The rationale behind this approach was that inactivating Cbl-b reduces the activation threshold for T cells and simultaneously decreases their sensitivity toward the suppressive effects of TGFβ. This should theoretically improve the efficacy of established checkpoint inhibition therapies.

In this study we confirm that loss of *Cblb* delays tumor growth and prolongs survival in a melanoma mouse model. Additionally, blocking CTLA-4 with a monoclonal antibody significantly boosts these effects. In contrast, however, inhibition of PD-L1-triggered signaling in *Cblb*-deficient mice shows no additive therapeutic benefit. Further *in vitro* results show that *Cblb*^−/−^ T cells are less susceptible to PD-L1-mediated suppression of proliferation and IFNγ secretion. These findings suggest that Cbl-b might have a so far unappreciated role in the inhibitory PD-1 signaling pathway in T cells.

## RESULTS

### CTLA-4 and PD-L1 blockade in *Cblb*^−/−^ mice

Blockade of CTLA-4 and/or PD-1 has been shown to drastically diminish tumor growth and prolong survival in mice and patients in a T cell-dependent fashion [14, 40, 41]. As *Cblb*^−/−^mice are able to immunologically reject otherwise lethal tumor burdens [33–38], we wanted to investigate if this effect would be even more profound in combination with CTLA-4 or PD-L1 blockade. Therefore, we subcutaneously injected 5 × 10^5^ B16ova cells into wild-type or *Cblb*^−/−^ mice, treated them with anti-CTLA-4, anti-PD-L1 or IgG control antibodies and monitored tumor growth.

**Figure 1:**
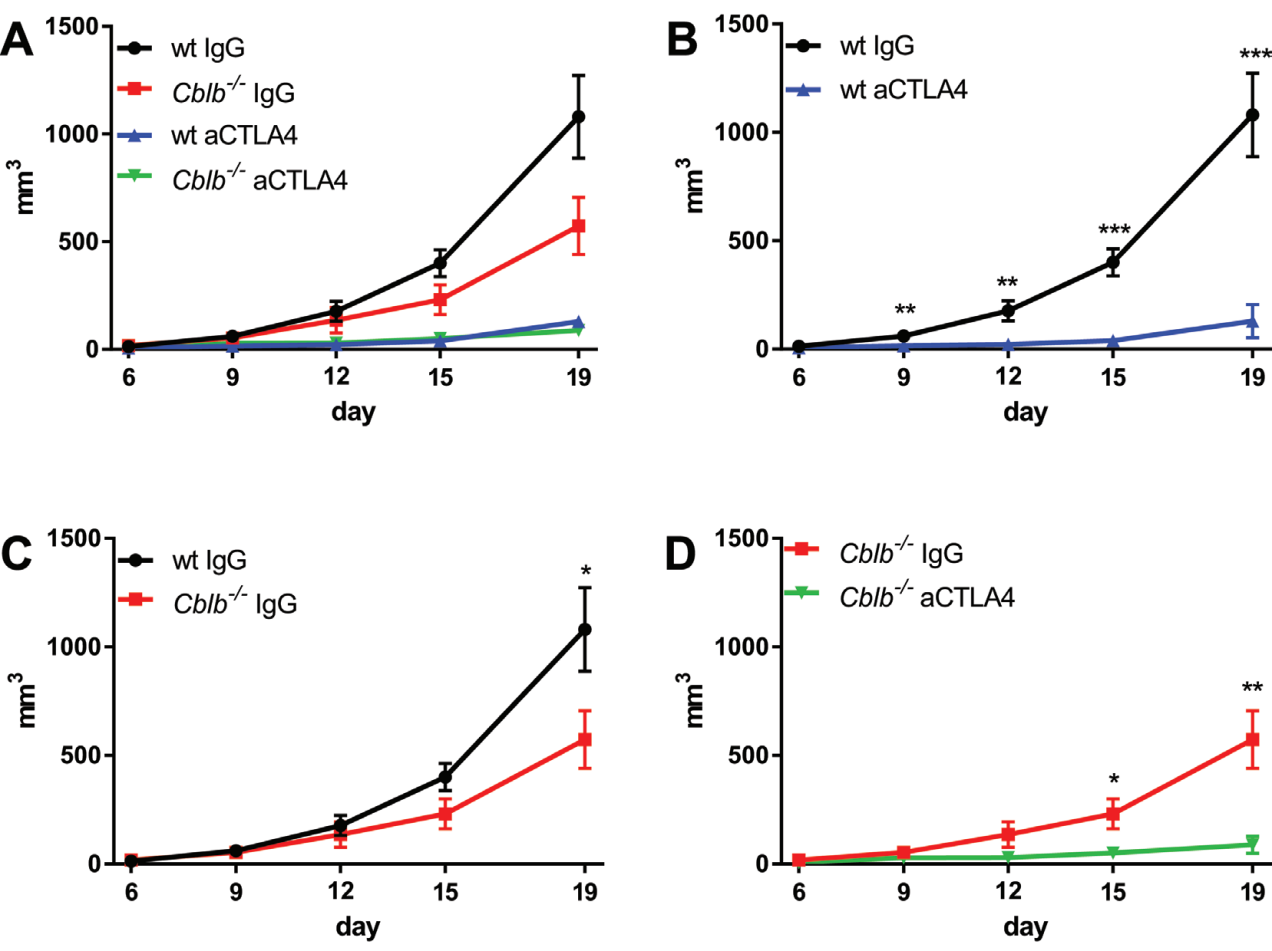
Kinetics of B16ova tumor growth C57Bl/6 (wt) and *Cblb−/−* mice were s.c. injected with 5×10^5^ B16ova cells and i.p. injected with 400μg anti-CTLA4 or IgG control antibody every 3rd day starting on day 0. The data are pooled from two independent experiments (*n* = 6-7 per group). Tumor volume is shown.

**Figure 2:**
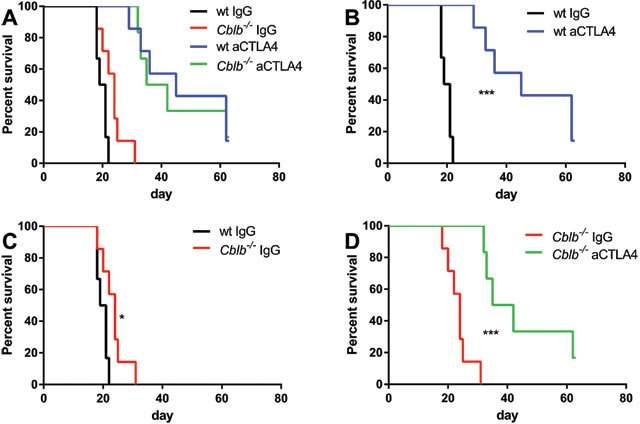
Survival percentages of mice from Figure 1 Mice were s.c. injected with 5×10^5^ B16ova cells and i.p. injected with 400μg anti-CTLA4 or IgG control antibody every 3rd day from day 0 until day 18. The data shown are pooled from two independent experiments (*n* = 6-7 per group).

As reported in previous studies, we found that CTLA-4 blockade or *Cblb* ablation led to a reduction of tumor growth (Figure 1A, 1B, 1C) and extended survival compared to wild-type IgG-treated mice (Figure 2A, 2B, 2C). Blocking of CTLA-4 in *Cblb*^−/−^ mice prolonged survival (Figure 2D) and attenuated tumor growth (Figure 1D) to an even more pronounced extent than in IgG-treated *Cblb*-deficient mice, suggesting that CTLA-4 signaling is not completely dependent on Cbl-b.

**Figure 3:**
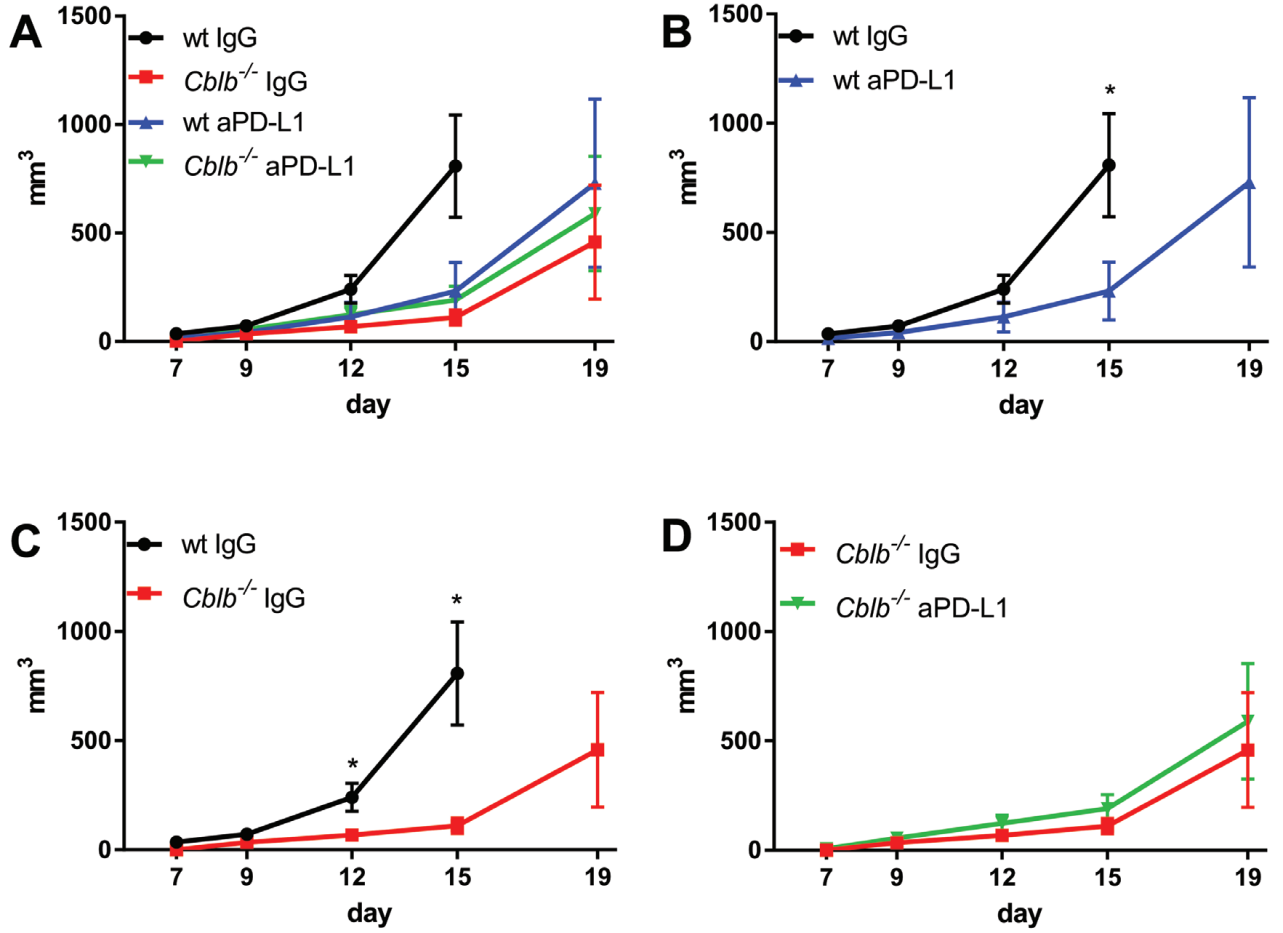
Kinetics of B16ova tumor growth C57Bl/6 (wt) and C*blb−/−* mice were s.c. injected with 5×10^5^ B16ova cells and i.p. injected with 500μg anti-PD-L1 or IgG control antibody every 3rd day starting on day 0. The data are pooled from two independent experiments (*n* = 6-8 per group). Tumor volume is shown.

**Figure 4:**
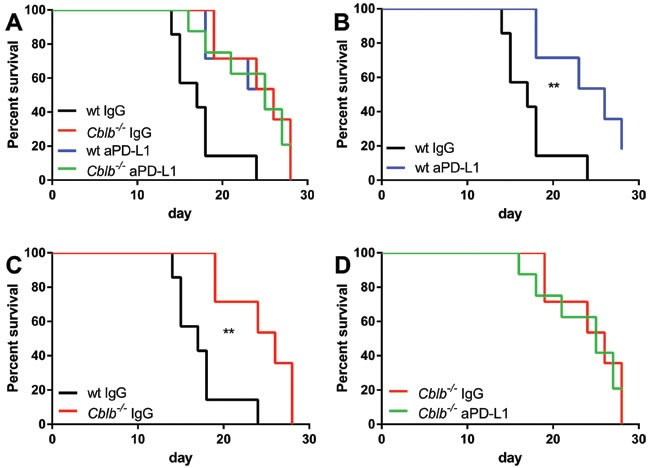
Survival percentages of mice from Figure 3 Mice were s.c. injected with 5×10^5^ B16ova cells and i.p. injected with 500μg anti-PD-L1 or IgG control antibody every 3rd day from day 0 until day 18. The data shown are pooled from two independent experiments (*n* = 6-8 per group).

As expected, treatment of wild-type mice with anti-PD-L1 significantly reduced tumor growth (Figure 3A, 3B) and prolonged survival (Figure 4A, 4B). Surprisingly, however, blocking of PD-L1 in *Cblb*^−/−^ mice had no additional effect (Figure 3D, Figure 4D). The anti-PD-L1-treated wild-type group behaved approximately the same as the *Cblb*^−/−^ IgG and *Cblb*^−/−^ anti-PD-L1 group (Figure 3A, Figure 4A). This suggests that PD-L1-initiated signaling is already impaired in *Cblb*-deficient mice.

### Distribution of PD-1 and CTLA-4 on T cells

**Figure 5:**
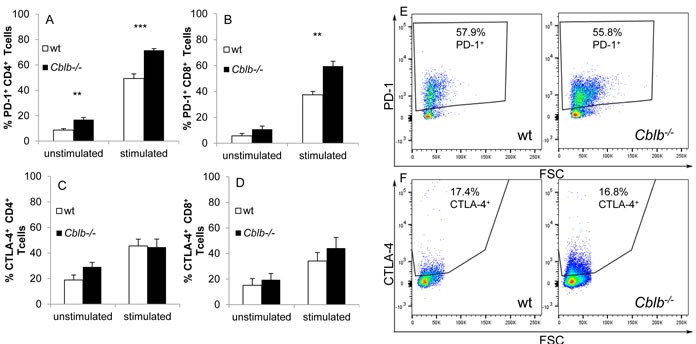
FACS analysis of T cells **A**.-**D**. CD3^+^ T cells of C57Bl/6 (wt) or *Cblb−/−* mice were cultivated overnight, either unstimulated or stimulated with 3μg/ml platebound anti-CD3 and 1μg/ml soluble anti-CD28. FACS analysis of CTLA-4 (C, D) and PD-1 (A, B) expression was performed on gated CD4^+^ (A, C) or CD8^+^ (B, D) cells. Mean +/− SEM of 4-6 individual mice is shown (3 independent experiments). **E**, **F**. PD-1 and CTLA-4 expression on tumor infiltrating CD8^+^ T cells. Dotblots are gated on CD45^+^ and CD8^+^ cells and percentage of PD-1^+^ or CTLA4^+^ cells is shown. One out of ten mice (four independent experiments) is depicted for each genotype.

In principle, the lack of any effect of blocking PD-L1 in *Cblb*-deficient mice could be due to diminished PD-L1 and/or PD-1 expression. To test this possibility, we performed FACS analysis of *in vitro* stimulated CD4^+^ and CD8^+^ T cells. PD-L1 (not shown), PD-1 and CTLA-4 expression on CD4^+^ and CD8^+^ T cells was induced upon CD3/CD28 stimulation (Figure 5). PD-1 expression on *Cblb*^−/−^ CD4^+^ and CD8^+^ T cells was significantly increased compared to wild-type cells (Figure 5A, 5B), whereas CTLA-4 seemed to be elevated only in CD8^+^ cells (Figure 5C, 5D). We did not detect any difference in the expression of PD-L1 in either of the genotypes (data not shown).

In four independent B16ova *in vivo* experiments without antibody treatment, PD-L1, PD-1 and CTLA-4 were expressed on tumor-infiltrating CD4^+^ and CD8^+^ cells, and no significant differences between wild-type and *Cblb* knockout mice were detected: wt CD8^+^ PD-1^+^: 56.0% ± 5.7%; *Cblb*^−/−^ CD8^+^ PD-1^+^: 50.3% ± 4.5%; wt CD8^+^ CTLA-4^+^: 17.1% ± 4.5%; *Cblb*^−/−^ CD8^+^ CTLA-4^+^: 18.0% ± 4.2% (*n* = 10) (Figure 5E, 5F and data not shown).

Furthermore, PD-L1 was expressed on a large fraction of B16ova tumor cells (wt: 70.3% ± 4.6%; *Cblb*^−/−^: 84.7% ± 4.3%), whereas PD-L2 was barely detectable (wt: 0.3% ± 0.1%; *Cblb*^−/−^: 0.6% ± 0.2%) (*n* = 6).

Interestingly, *Cblb*-deficient tumor infiltrating antigen presenting cells (CD45^+^ MHC-II^+^) expressed significantly more PD-L1 on a per cell basis (not shown) and also the fraction of PD-L1 positive cells was significantly higher (wt: 85.7% ± 1.5%; *Cblb*^−/−^: 92.3% ± 1.9%) (*p* = 0.035; *n* = 6).

### *Cblb*-deficient T cells are less susceptible to PD-L1-mediated suppression

**Figure 6:**
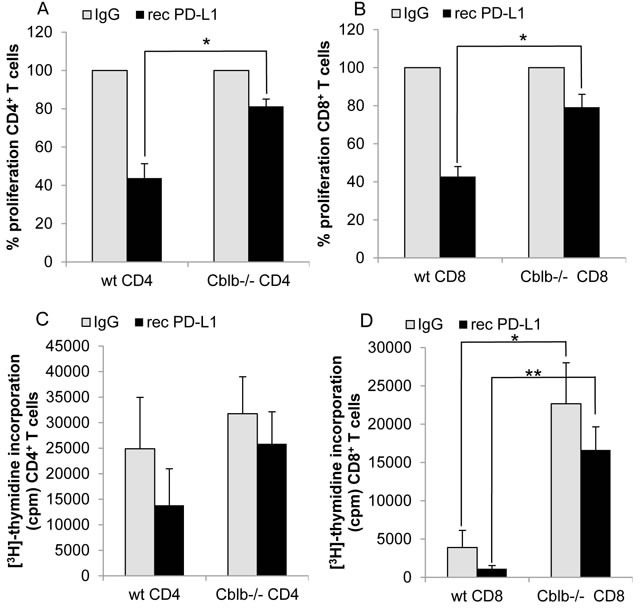
Proliferation of T cells CD4^+^ **A**., **C**. or CD8^+^ **B**., **D**. T cells were stimulated with 1μg/ml platebound anti-CD3 and 0.1μg/ml soluble anti-CD28. Additionally, wells were coated with 10μg/ml recombinant PD-L1 or IgG as control. [^3^H]-thymidine was added on day 2 of cell culture and its incorporation measured after 16h. A, B: Control value was set to 100%; C, D: Counts per minute of [^3^H]-thymidine incorporation. Mean ± SEM of 4-5 individual mice is shown (3 independent experiments).

**Figure 7:**
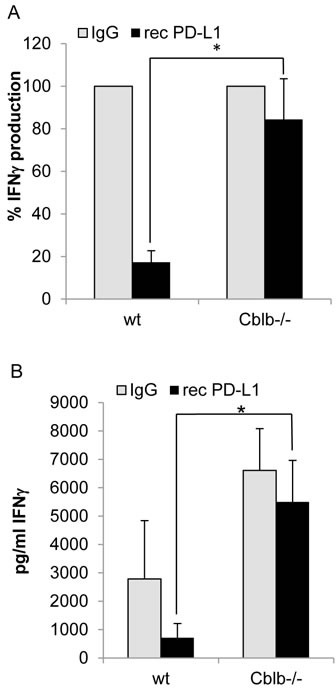
IFNγ production of CD8 **^+^** T cells. Cells were stimulated with 1μg/ml platebound anti-CD3 and 0.1μg/ml soluble anti-CD28. Additionally, wells were coated with 10μg/ml recombinant PD-L1 or IgG as control. Supernatants were taken on day 2 of cell culture and IFNγ was measured with Bioplex technology. A: Control value was set to 100%; B: Concentration in the supernatants. Mean ± SEM of 6-7 individual mice is shown (4 independent experiments).

Since we did see an effect of anti-PD-L1 treatment on tumor growth and survival in wild-type but not *Cblb*^−/−^ mice (Figure 3A), we speculated that Cbl-b could play a role in the suppressive PD-1 signaling pathway. To test this hypothesis, we performed *in vitro* inhibition assays using recombinant PD-L1, immobilized on the surface of culture plates. Proliferation of both CD4^+^ and CD8^+^ cells was substantially diminished by active PD-1 signaling triggered by recombinant PD-L1. However, proliferation of wild-type cells was downregulated to approximately 44% of the uninhibited control value, whereas *Cblb*^−/−^ cells still showed a proliferative response of 80%, indicating that *Cblb*-deficient T cells were hyporesponsive to PD-L1-mediated suppression of proliferation (Figure 6A, 6B). Moreover, expression of the pro-inflammatory anti-tumor cytokine IFNγ was strongly reduced in wild-type CD8^+^ cells (down to 17%) but not in *Cblb* knockout CD8^+^ cells (down to 85%) (Figure 7). These results suggest that Cbl-b partly mediates the suppressive effects of PD-1 engagement. Along this line, it was reported that silencing PD-L1 in antigen presenting dendritic cells led to diminished Cbl-b protein levels in T cells, suggesting an involvement of PD-1 signaling in upregulation of Cbl-b expression [47]. However, in our experimental system we did not observe enhanced Cbl-b protein amounts after stimulation of T cells with recombinant PD-L1 (Supplementary Figure 3).

## DISCUSSION

Immunotherapies against malignant neoplastic diseases emerge more and more as a promising “fifth pillar” of cancer treatment (besides surgery, radiation therapy, chemotherapy, and molecularly targeted therapies) [48]. The great expectations raised by this kind of treatment are underlined by the decision of the journal “Science” to choose cancer immunotherapy as “Breakthrough of the Year” in 2013 [49].

The rationale behind immunotherapies is to harness the immune system's ability to eliminate structures that are recognized as “foreign”. In the case of malignant disease, these structures turn out to be cancer-associated antigens that are capable of eliciting an immune response, most notably activating cytotoxic T lymphocytes via their specific antigen receptors [50].

Unfortunately, however, T cell activity towards tumors is often compromised by inhibitory signals, the so-called “immune checkpoints”. Consequently, an important aspect of cancer immunotherapy is to block these inhibitory signals in an effort to unleash T cells to destroy malignant cells.

The E3 ubiquitin ligase Cbl-b has been shown to act as an intracellular checkpoint by interfering with a number of T cell signaling pathways [51]. Genetic loss of *Cblb* leads to anergy resistance and susceptibility to autoimmunity [27, 28, 52]. Cbl-b also contributes to the maintenance of self-tolerance by mediating the immunosuppressive effects of TGFβ [32]. In keeping with these observations, *Cblb* knockout mice reject tumors, which has been shown to crucially depend on the CD8^+^ T cell compartment [30, 34, 36, 38].

As proof of concept that Cbl-b may serve as a novel cancer immunotherapy target, we have already shown that adoptive transfer of *Cblb*-silenced polyclonal CD8^+^ T cells together with DC vaccination resulted in strong suppression of tumor growth with substantially prolonged overall survival, interestingly without any sign of autoimmunity [35]. Recently, it has been shown in a phase I trial that the strategy of silencing *Cblb* in human peripheral blood mononuclear cells followed by adoptive cell transfer is feasible and safe [53].

Considering the complexity of T cell signaling pathways, combining the inhibition of distinct immune checkpoints presumably has the benefit of targeting key regulators at different cellular levels. For example, patients with advanced melanoma show a response rate of 11% with ipilimumab (anti-CTLA-4), which is increased up to 61% when treated in combination with nivolumab (anti-PD-1) [42]. Several additional combinatorial treatments are of particular interest, such as blockade of PD-1 together with LAG-3 or TIM-3 [54, 55].

In this study, we tested the effectiveness of the strategy of inhibiting the established immune checkpoints CTLA-4 (via CTLA-4 neutralization) or PD-1 (via neutralization of the PD-1 ligand PD-L1) in combination with targeting the intracellular checkpoint Cbl-b in order to enhance T cell anti-tumor activity in a B16ova melanoma mouse model (B16ova expresses PD-L1, not shown). In wild-type mice, CTLA-4 inhibition showed a marked efficacy in terms of decreased tumor growth (Figure 1B) and prolonged survival (Figure 2B) compared to IgG-treated control mice. Similarly, anti-CTLA-4 treatment also had a significant effect in *Cblb* knockout mice (Figure 1D & 2D). Neutralizing PD-L1 with a monoclonal antibody also delayed tumor growth and extended survival, albeit to a lesser extent than CTLA-4 inhibition (Figure 3B & 4B). It is noticeable that in our murine melanoma model, anti-PD-L1 treatment is less effective than anti-CTLA-4 treatment even in wild-type mice. In the clinical context, blocking PD-L1/PD-1 interaction with pembrolizumab is more effective than blocking CTLA-4 with ipilimumab in the treatment of advanced melanoma [56]. This apparent discrepancy could be possibly explained by the different species, materials, and methods used. For example, the neutralizing antibodies in our study are completely different from the clinically applied immunoglobulins.

Surprisingly, anti-PD-L1 treatment was completely ineffective in *Cblb* knockout mice (Figure 3D & 4D), which is in striking contrast to CTLA-4 neutralization. This was not simply due to diminished PD-1 receptor expression, because PD-1 amounts were even enhanced in *Cblb*-deficient CD4^+^ and CD8^+^ T cells *in vitro* (Figure 5A & 5B) or not significantly altered on *Cblb*-deficient tumor infiltrating T cells, respectively (Figure 5E).

In the PD-L1 neutralization tumor experiments, the three groups “wild-type anti-PD-L1”, “*Cblb*^−/−^ IgG”, and “ *Cblb*^−/−^ anti-PD-L1” exhibited virtually identical benefits in terms of tumor growth and survival rates compared to the “wild-type IgG” control group (Figure 3A & 4A), possibly reflecting an involvement of Cbl-b in the PD-1 signaling pathway. However, this is not a definite proof that Cbl-b is downstream of PD-1, but this assumption is supported by *in vitro* inhibition assays using recombinant PD-L1. PD-L1 potently reduced the proliferation of CD4^+^ and CD8^+^ T cells (56% and 57% reduction, respectively), whereas it was much less effective in *Cblb*-deficient T cells (19% and 21% reduction). Furthermore, PD-1 engagement strongly impaired IFNγ secretion of wild-type CD8^+^ T cells (83% reduction), in striking contrast to *Cblb* knockout cells that were almost resistant to recPD-L1 (16% reduction). These results indicate that Cbl-b substantially mediates the suppressive effects of PD-L1/PD-1 signaling, suggesting a biochemical function of Cbl-b downstream of PD-1. Of note, during this manuscript was under revision, a paper was published confirming that *Cblb*-deficient T cells (and NK cells) are resistant to PD-L1/PD-1-mediated suppression [57].

Cbl-b amounts were reported to be diminished in T cells upon blockade of PD-1/PD-L1 interaction [47]. Along this line, SHP-1 and SHP-2 are recruited to PD-1 [58], and SHP-1 dephosphorylates and stabilizes Cbl-b [59]. It has also been shown that PD-1 ligation impairs PKCθ activation loop phosphorylation [60]. Impaired PKCθ activity also prolongs the half-life of Cbl-b, given that Cbl-b degradation is dependent on PKCθ [61].

Taken together, our findings suggest a novel function of Cbl-b in the PD-1 signaling pathway in effector T cells. However, whether Cbl-b exerts a specific function as an E3 ligase in the PD-1 signaling pathway remains speculative and warrants further investigation. Unraveling the detailed signaling mechanisms of the non-redundant role of Cbl-b in the PD-1 function of T cells in future studies may contribute to an improved understanding of the molecular and cellular processes that lead to autoimmunity or cancer.

## MATERIALS AND METHODS

### Mice

*Cblb* knockout mice on a C57Bl/6 background were described previously [27]. Mice were maintained under SPF conditions. All animal experiments were performed in accordance with the Austrian “Tierversuchsgesetz” (BGBI. Nr.501/1989 i.d.g.F. and BMWF-66.011/0061-II/3b/2013) and were approved by the Bundesministerium für Wissenschaft und Forschung (bm:wf).

### Tumor induction and *in vivo* antibody administration

8- to 12-week-old female mice were injected subcutaneously with 100μl PBS containing 5×10^5^ B16ova melanoma cells into the left, ventral flank. Intraperitoneal injection of 200μl PBS containing either 0.4mg of a Syrian hamster anti-mouse CTLA4 (Clone UC10-4F10-11; BE0131), 0.5mg rat anti-mouse PD-L1 (Clone10F.9G2; BE0101), 0.4mg Syrian hamster IgG (BE0087) or 0.5mg rat IgG2b (Clone LTF-2; BE0090) (all from BioXCell, USA) was performed every 3 days starting from day 0 of B16ova challenge until day 18.

Tumor volume was calculated according to the following equation: V = π/6*length*width^2^. For survival analysis, mice with tumors exceeding the length limit of 15 mm were sacrificed and counted as dead.

### Flow cytometry

Spleen and lymph nodes of 4-6 mice per genotype (not pooled) were mashed through a sieve, depleted of erythrocytes using the mouse erythrocyte lysing kit (R&D, WL2000) and CD3^+^ T-cells were isolated untouched using the Pan T Cell Isolation Kit II mouse (Miltenyi Biotech 130-095-130) according to manufacturer's protocol.

750.000 cells per 96 flatbottom well were cultured overnight in 300μl RPMI+++ (Roswell Park Memorial Institute 1640 medium [PAA; E15-039] supplemented with 10% fetal calf serum [FCS], 2 mM l-glutamine and penicillin-streptomycin [50 U/ml]) and left unstimulated or stimulated with 3μg/ml platebound anti-CD3 (2C11, in-house made) and 1μg/ml soluble anti-CD28 (BD 553294).

Cells were harvested the next day, fixed and permeabilized (buffers from the FoxP3 staining buffer set, eBiosciences 00-5523), before staining for 20min at 4°C with specific antibodies (CD4 FITC, BD 553047; PD-1 PE, eBiosciences 12-9985-82; PD-L1 PE-Cy7, eBiosciences 25-5982-20; CTLA-4 APC, eBiosciences 17-1522-80; CD8 FITC, eBiosciences 11-0081-85), diluted 1:200 in permeabilization buffer (eBiosciences 00-8333). Data acquisition was performed on a FACSCalibur and data analysis was conducted using the FlowJo 10.0.8r1 software.

For analysis of tumor infiltrating lymphocytes, 8- to 12-week-old female mice were injected subcutaneously with 100μl PBS containing 1×10^5^ B16ova melanoma cells into the left, ventral flank. The mice were controlled regularly and when the first tumor exceeded the length of 12mm the tumors of all mice were collected on the same day (four independent experiments: between day19 and day 22 after injection). The tumors were cut in small pieces and digested for 45 min in PBS containing 2% FCS, 2.5mM MgCl, 2.5 mg/ml collagenase D (Roche, 11088858001) and 1 mg/ml DNase I (Roche, 11284932001) at 37°C. Digested tissues were incubated 5 min at 37°C with EDTA (0.5 M) to prevent DC/T cell aggregates and mashed through a 70-μm filter and a 40-μm filter. Cells were washed, fixed, permeabilized (buffers from the FoxP3 staining buffer set, eBiosciences 00-5523) and stained for 20min at 4°C with specific antibodies (PD-1 PE, eBiosciences 12-9985-82; PD-L1 PE-Cy7, eBiosciences 25-5982-80; PD-L2 FITC, eBiosciences 11-9972-82; CD8 PerCP-Cy5.5, eBiosciences 45-0081-80; CD4 V500 BD 560782; CD45 APC-Cy7, BD 561037; CTLA-4 APC, eBiosciences 17-1522-80) diluted 1:200 in permeabilization buffer (eBiosciences 00-8333). Data acquisition was performed on a FACSVerse and data analysis was conducted using the FlowJo 10.0.8r1 software.

### Analysis of proliferative responses and cytokine release

96well flatbottom wells were coated with PBS containing 10μg/ml IgG1 (HRPN; BE0088 BioXcell) or recombinant mouse PD-L1 Fc chimera protein (R&D, 1019-B7) at 37°C over night. This solution was then discarded and replaced by PBS containing 1μg/ml anti-CD3 (2C11, in-house made) and left for 4h at 37°C. After coating, 400.000 CD4^+^ or CD8^+^ cells (negative MACS sort of spleen and lymph nodes according to manufacturer's protocol; Milteny 130-104-454 and 130-104-075) per well were cultured for 3 days in 200μl RPMI+++ and costimulated with 0.1μg/ml anti-CD28 (BD 553294). On day 2 of culture, 50μl supernatants were taken for measurement of cytokines via BioPlex technology (BioRad) and 50μl RPMI+++ containing 1 μCi [^3^H]-thymidine (Perkin Elmer) were added. After a 16-hour pulse, cells were harvested and [^3^H]-thymidine incorporation was measured with a Matrix 96 direct β counter system.

### Western blot

Wells of a 24 well plate were coated with PBS containing 10μg/ml IgG1 (HRPN; BE0088 BioXcell) or recombinant mouse PD-L1 Fc chimera protein (R&D, 1019-B7) at 37°C overnight. This solution was then discarded and replaced by PBS containing 1μg/ml anti-CD3 (2C11, in-house made) and left for 3h at 37°C. After coating, 1×10^7^ wild-type CD3^+^ cells (negative MACS sort of spleen and lymph nodes according to manufacturer's protocol; Milteny 130-095-130) per well were cultured overnight in 1ml RPMI+++ and costimulated with 1μg/ml anti-CD28 (BD 553294). Cells were collected, washed once in ice-cold PBS and lysed in 30μl lysisbuffer (5mM NaP_2_P, 5mM NaF, 1mM Na_3_VO_4_, 5 mM EDTA, 150mM NaCl, 50mM tris [pH 7.3], 1% NP-40, aprotinin and leupeptin [50μg/ml each]) for 30min on ice. After centrifugation (15.000g, 4°C), protein lysates were subjected to Western blotting analysis with antibodies against Cbl-b (abcam 54362; dilution 1:1000) and actin (santacruz 1615; dilution 1:1000). Densitometric analysis was performed using ImageJ.

### Statistics

Results are expressed as mean ± standard error of the mean (SEM). Groups were compared using the paired Student's *t*-test. Overall survival was expressed using the Kaplan-Meier method and differences between groups were determined using the log-rank test. Data analysis was performed using GraphPad Prism 7.00. Significant differences are indicated as *P ≤ 0.05, **P ≤ 0.01, and ***P ≤ 0.001.

## SUPPLEMENTARY MATERIALS AND FIGURES


