# Supplementary material for: Cblb-deficient T cells are less susceptible to PD-L1-mediated inhibition
Source: Oncotarget. 2017 Jun 3;8(26):41841–53. doi: 10.18632/oncotarget.18360 (PMC5522032; doi:10.18632/oncotarget.18360)
Supplement: Supplementary file 1 [file oncotarget-08-41841-s001.pdf]

# *Cblb*-deficient T cells are less susceptible to PD-L1-mediated inhibition

## Supplementary Material

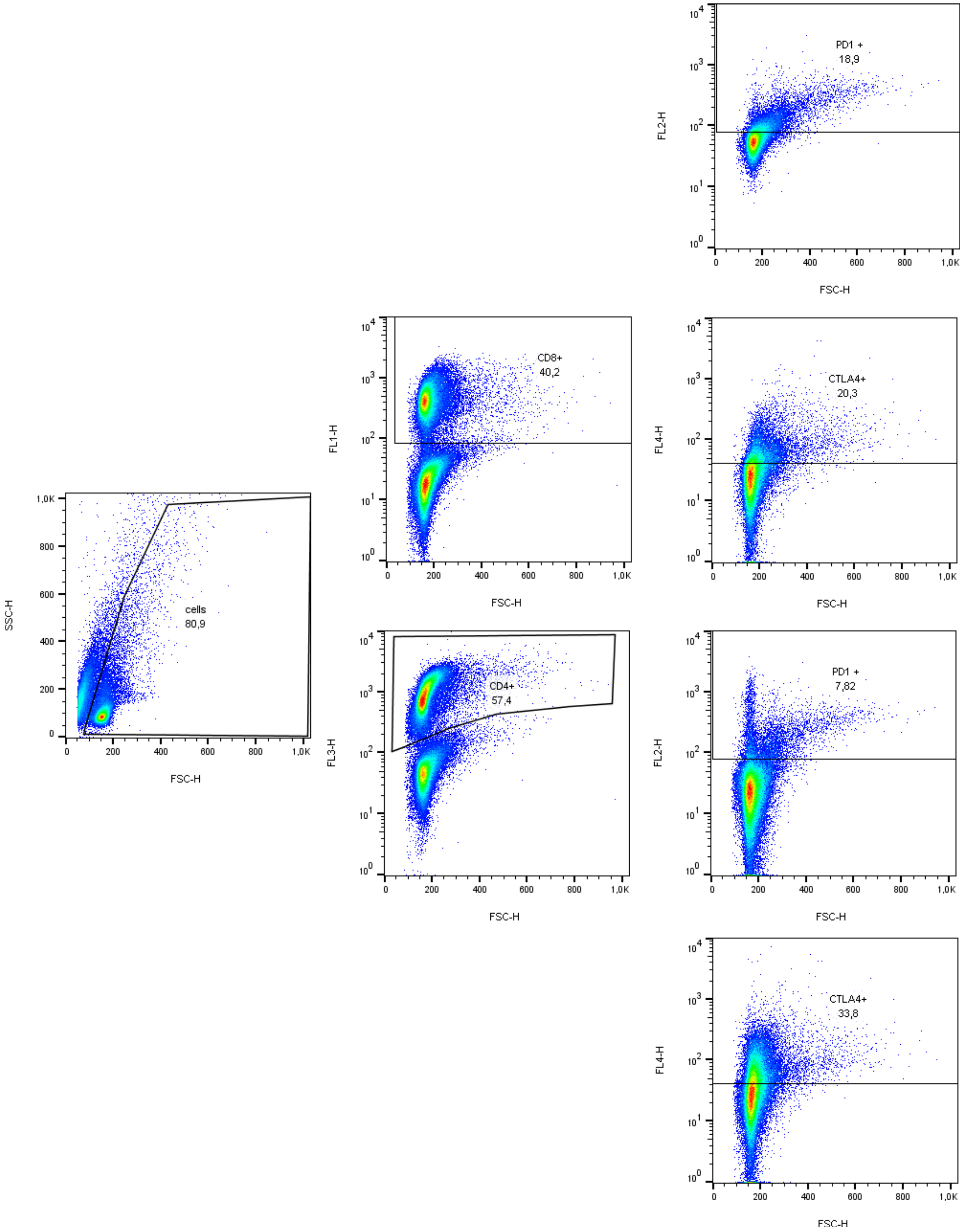


**Supplementary Figure 1: Gating strategy of the results presented in figures 5A-D.** FL1: CD8 FITC; FL2: PD-1 PE; FL3: CD4 PeCy7; FL4: CTLA-4 APC

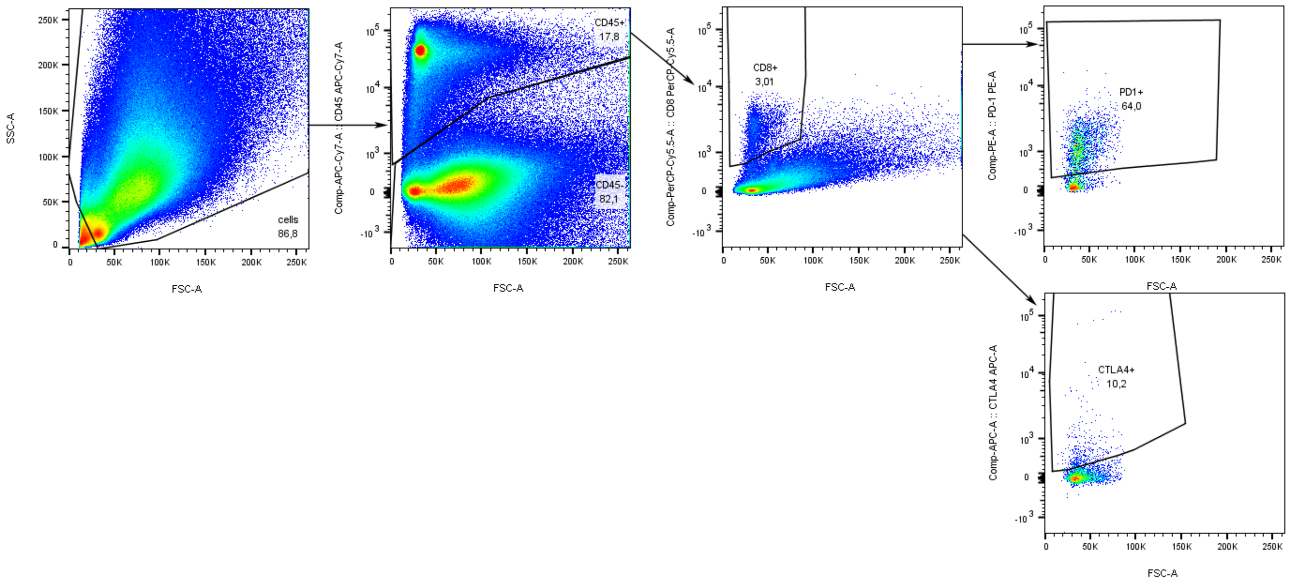


**Supplementary Figure 2: Gating strategy of the results presented in figures 5E, F.**

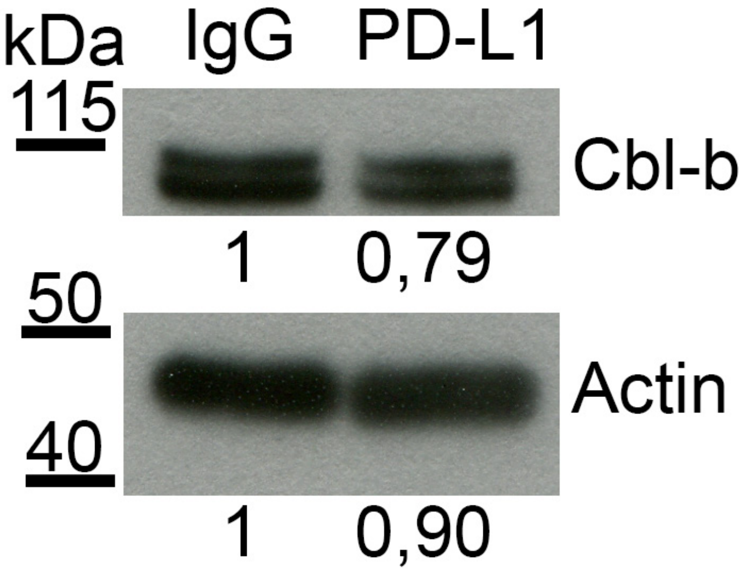


**Supplementary Figure 3: Western blot analysis of Cbl-b in T cells treated with or without PD-L1.** $CD3^+$ cells were stimulated overnight with 1µg/ml plate-bound anti-CD3 and 1µg/ml soluble anti-CD28. 10µg/ml recombinant PD-L1 or control IgG was added as indicated. One of two experiments with similar results is shown. Actin served as a loading control. Numbers indicate relative expression levels.
